# Professional Boundaries and Ethical Obligations in Saudi Arabia An Integrated Sharia–Saudi Legal–Clinical Framework

**DOI:** 10.12688/f1000research.178561.1

**Published:** 2026-04-01

**Authors:** Hajed A. Alotaibi, Motaz T. Alotaibi

**Affiliations:** 1 Department of Sharia, College of Sharia and Law, Majmaah University, Al Majmaah, 11952, Saudi Arabia; 2 Department of Psychology, College of Social Sciences, Imam Mohammad Ibn Saud Islamic University, Riyadh, Saudi Arabia, Saudi Arabia

**Keywords:** Sharia; Saudi Arabia; clinical ethics; telepsychology; data protection

## Abstract

**Background:**

Professional boundaries in psychotherapy are structural safeguards within a fiduciary, power-imbalanced relationship. In Saudi Arabia, boundary dilemmas arise in a Sharia-informed moral environment and a regulated healthcare system, commonly involving mixed-gender privacy sensitivities, gifts and culturally normative hospitality, dual relationships in close-knit communities, conflicts of interest, and technology-mediated challenges (e.g., messaging platforms and tele-mental health).

**Methods:**

This study uses a secondary-data integrative design combining (i) doctrinal analysis of publicly available Saudi regulatory and professional materials relevant to professional conduct and accountability (healthcare practice governance, professional ethics standards, anti-harassment protections, and personal data governance), (ii) normative Sharia reasoning grounded in established ethical concepts and juristic method (amānah/trust, dignity protection, harm prevention, and avoidance of risk-pathways), and (iii) synthesis of peerreviewed clinical ethics literature on boundary crossings versus violations, patient harm, and risk management (consultation, supervision, documentation, and institutional policy). No participant recruitment, surveys, interviews, vignettes, or clinical record data were used.

**Results:**

The analysis shows convergence across Sharia ethics, Saudi regulatory governance, and clinical ethics scholarship on a coherent model: boundaries should function as the default protective structure, while limited boundary crossings may be defensible only when clinically justified, proportionate, transparent, and documented. The paper operationalizes this model through a Saudi-tailored Code of Conduct, decision rules for recurring dilemmas (mixedgender safeguards, gifts/hospitality, dual relationships and conflicts of interest, exploitation of dependency, and digital contact), and governance recommendations for training, supervision pathways, documentation templates, safe reporting, and secure communication policies aligned with data protection expectations.

**Conclusions:**

An integrated Sharia–Saudi legal–clinical boundary framework strengthens patient dignity, reduces exploitation risk, supports defensible clinical decision-making, and promotes public trust in mental health services in Saudi Arabia during rapid digital transformation.

## 1. Introduction

Professional boundaries do not consist of etiquette or good manners in psychotherapy. They are a formal protection which specifies the therapeutic relationship as professional, fiduciary, and time-limited, and not social, commercial, romantic, or familial. Fiduciary relationships involve the specialisation of knowledge and institutional power, and the access to very sensitive information on the part of one party. The other party (the client/patient) will frequently join the relationship in a vulnerable, distressed, dependent, or less self-defending state. The power wielded by the clinician must only be utilised in the best interest of the client and never against personal interests, so the frame of the therapy- setting with its clarity of role, rules of confidentiality, schedule, limits of payment and limit of contact, etc. are useful in ensuring that the power of the clinician is not abused ( Gutheil & Gabbard, 1993). Clinically, boundaries safeguard the integrity of treatment in at least three overlapping spheres. To begin with, they maintain role clarity, which reduces confusion about what the therapist is there for, what the client can realistically expect, and what the relationship is not ( Gutheil & Gabbard, 1993). Second, they address the issue of power asymmetry.

Asymmetrical disclosure in therapy occurs when clients share confidential information with therapists, who tend to share selectively and strategically. This asymmetry is clinically required, and it also poses the threat of ethical vulnerability when the therapist starts using the client emotionally, socially, sexually and financially ( Barnett et al., 2007). Third, boundaries safeguard against expected distortions in the therapeutic relationship, such as transference and countertransference. The clients can become strongly attached, idealise, fear of being abandoned, or want to please, the therapists can also have rescue fantasies, over-identification and unfulfilled personal needs. Once demarcations become fragile, these relations may turn into clinically serviceable content and channels to abuse or maltreat ( Gutheil & Gabbard, 1993; Smith & Fitzpatrick, 1995).

Professionally, contemporary professional codes consider boundary management an essential responsibility of the clinician, grounded in the requirements of beneficence, nonmaleficence, and fidelity. The code of ethics by the American Psychological Association specifically covers various relationships/conflicts of interest as well as forbidding exploitative relationships, such as sexual relationships with current therapy clients ( American Psychological Association, 2017). This is not only moral idealism but also a risk-management fact: psychological treatment can create a sense of dependency and oversensitivity to relationships, and so-called mutually beneficial relationships prove morally invalid due to the power imbalance and the client’s therapeutic helplessness ( Barnett et al., 2007; Gottlieb & Younggren, 2009).

One point of analysis that would be helpful to publish and to develop the policy used by Saudi policymakers is the confusion between boundary crossings and boundary violations. Boundary crossings are departures from normal therapeutic parameters that can be clinically, culturally, and time-constrained when they are unmistakably in the client’s best interest and do not pose a threat of exploitation ( Smith & Fitzpatrick, 1995). Cases in the literature include thoughtful attendance at a culturally significant event in exceptional situations, a little supportive contact to normalise an acute risk if clinically warranted, or a nominal culturally normative gift post-termination ( Smith & Fitzpatrick, 1995). Boundary violations, by contrast, are destructive, exploitative or therapist-serving departures that predictably harm the client, distort the therapy or transform the relationship into another role (i.e. sexual contact, coercion, humiliation, recurring dual-role entanglements, financial exploitation or creating dependency to meet his emotional needs) ( Gutheil & Gabbard, 1993). Notably, the ethical issue is not change as such but direction and role: would the action safeguard the client and treatment, or would it redirect gratification, power, or advantage to the clinician?

The slipping slope issue is clinically significant and should be approached with subtlety. The literature cautions that minor, normalised boundary violations would gradually undermine judgment, particularly in situations of stress, isolation, burnout, or poor oversight, since each step would make the next seem exceptional ( Gottlieb & Younggren, 2009). Nevertheless, ethical analysis also accepts that not all boundary crossing necessarily results in violation: what is important is that the clinician is using disciplined safeguards: clear clinical rationale, proportionality, consultation/supervision, and documentation ( Gottlieb & Younggren, 2009; Barnett et al., 2007). When it comes to the publishable operational terms, the question is whether the clinician can show that the departure is client-benefiting, non-exploitative and low-risk now and in the long term? When that is not justifiable, the most ethical position is not to cross.

One of the most impactful areas of ethical concern in psychotherapy is boundary failure, as it is capable of inflicting direct harm (psychological harm, retraumatisation, shame, coercion), long-term damage to trust, and damage to the system on the level of community confidence in mental health services. One especially serious group of them is sexual boundary violations and grooming-like dynamics that may cause very serious and lasting damage and are generally accepted as incompatible with ethical psychotherapy by jurisdiction ( Gutheil & Gabbard, 1993; Hook & Devereux, 2018). There is also a dropout, increased symptoms, and resistance to seeking future assistance, which can be caused by even non-sexual violations, such as entanglement in finances, persistent personal revelations that turn the roles around, and dependency that is manipulative. This is why ethics advice sets the scope of boundaries within the patient safety infrastructure rather than the therapist’s style.

Possible reasons that lead to the intensification of boundary risks in Saudi Arabia (Sharia legal-clinical integration).

There are no country-specific aspects to the professional boundary dilemma, but a combination of interacting factors in the Saudi situation can increase the risk of boundary strain and the severity of damage from boundary drift. The awareness of such context variables enhances the paper’s originality, as it transcends general ethical principles to operationalise Saudi-relevant risk management and governance.

1) **Moral environment based on Sharia: amana and dignity, and eschewing morally risky circumstances.**
  Saudi clinical practice lies within a moral field where trustworthiness (amah) and the defence of dignity are not just individual values but a socially pertinent expectation of professional practice, particularly in healthcare ( Chamsi-Pasha & Albar, 2016; Daar & al Khitamy, 2001). This is important in psychotherapy, as clients often disclose intimate and socially consequential information. In such a case, where the clinician’s role is perceived as too close, too informal, or even too secretive, the client and family may view the clinician in a moral rather than clinical light. The risk of such an interpretation may heighten suspicion, lead to misconceptions, raise reputational issues, and, crucially, result in premature termination, despite the clinician’s supporting motive.
  The second amplifier is that cross-gender professional interaction can heighten sensitivities to modesty norms, privacy, physical contact, and seclusion issues in particular settings. Islamic bioethics research observes that interactions across gender may affect patient comfort and expectations, and may define respectful, safe, or ethically acceptable practices for the patient ( Padela & Rodriguez del Pozo, 2011). It is not that clinical point that psychotherapy ought to be a gender-rule exercise, but that boundary clarity should be culturally intelligible. For example, what is referred to as neutral friendliness in Western ethics can be perceived as a special relationship in a local context, provided that there is unstructured disclosure of self, texting via personal devices, and unusual, flexible meeting schedules.
  This is the point at which Sharia-informed reasoning can be of practical use: it does not focus solely on the avoidance of harm, but also on avoiding foreseeably dangerous means to harm (often called, in juristic method, blocking means to wrongdoing, sadd al-dharā’iʿ; Kamali, 2003). In terms of boundary, it would mean formulating professional encounters in a manner that they are transparent and role limited: clinic design which does not lead to unintentional seclusion, appointment protocol, clear communication policy, and documentation practices that minimise confusion. Stated differently, Sharia-informed ethics can be operationalised as preventive professionalism, which is a framework that safeguards the dignity of the client and integrity of the clinician Alotaibi, H. A. (2021).
  Lastly, because stigma is still a current issue of mental health help-seeking in the Saudi Arabian context, the social cost of the boundary confusion can be greater in comparison with the areas where the help-seeking practices linked to mental health care are more normalised. Clients might want to be exposed as little as possible and maintain maximum clarity about their role when they are afraid of shame, losing reputation, or experiencing conflict with a family ( Alhumaidan et al., 2024). That fact predisposes the idea of boundary governance to a problem of public trust: the more secure and organised the role, the less clients feel afraid to seek care without worrying about being socially misunderstood.
2) **The institutional accountability and professional regulation in Saudi Arabia.**
  The regulatory and institutional environment also increases the professional boundaries. The ethics of Saudi practice is informed by professional ethics, specifically the Saudi Commission for Health Specialities Code of Ethics for Healthcare Practitioners, which focuses on professionalism, respect, avoidance of misconduct, and safeguarding patients’ welfare ( SCFHS, 2014). These tools of ethics do not simply enumerate ideals: they establish a situation of discipline and accountability. As a result, boundary lapses can not only lead to clinical failures but also regulatory risks, influencing licensure status, institutional privileges, and professional reputation.
  Simultaneously, the healthcare practice regulation implemented by the Ministry of Health (Saudi Arabia) sets out general expectations for healthcare professionals regarding governance ( Ministry of Health, 2005). Although such instruments do not describe all psychotherapy cases, they affirm the main idea that professionals’ roles should not be used against patients’ best interests or against legal and ethical practice. These structures are shaped by institutional policies governing professional performance, complaint management, supervision, and documentation.
  On a larger scale within the workplace, this legislation is supported by the Kingdom of Saudi Arabia Anti-Harassment Law, which disapproves of exploitative or coercive conduct ( Kingdom of Saudi Arabia, 2018). Although the law of harassment has no specific relation to psychotherapy, it is of particular concern as the violations of the boundaries tend to be accompanied by the abuse of power and coercion. The legal environment, hence, favours a stringent enforcement of the boundaries as a harm-prevention tool, particularly against sexualised behaviour, intimidation or recurring unwanted touch.
3) **Boundary accelerating factor (tele-mental health and ubiquitous messaging) as a digital transformation.**
  DigitisationDigitisation contributes to the strain on boundaries, as contact is easy, regular, and casual. Texting and messaging applications may conflict with time boundaries (always available), teletherapy may conflict with place boundaries (sessions at people’s homes where a certain level of privacy is not guaranteed), and social media may accidentally establish dual relationships or unintentionally allow access to personal data. The clinical risk involved is that what at first seemed convenient turns out to be unorganised dependency - the client is taught that the reassurance can be received outside of the therapy. The therapist is taught that responding will reduce short-term anxiety, even though it will increase long-term dependency.
  Guidelines on ethics and practice regarding telepsychology state that remote services must be planned expressly with privacy, communication, documentation, emergencies, and role limits in mind ( Joint Task Force for the Development of Telepsychology Guidelines for Psychologists [JTF], 2013). Tele-contexts also present special challenges in terms of boundaries that demand proactive organisation rather than improvisation ( Drum & Littleton, 2014). In sum, digital boundaries should be regarded as central to professionalism rather than an isolated peripheral solution.
  Digitisation in Saudi contexts is also associated with personal data governance. The Saudi Data and AI Authority’s personal data protection framework anticipates that sensitive personal data (such as health data) should be processed with minimum restrictions, security measures, and authorised access ( SDAIA, 2023). When contact is clinically well-intentioned, the use of personal devices, unapproved applications, or undocumented messaging may expose privacy and compliance risks.
4) **Saudi ethics literature on healthcare signal.**
  In the Saudi context, there are no boundless situations that are theoretical. Empirical studies of ethical attitudes and awareness among Saudi clinicians indicate inconsistencies in the processes of appraisal of boundary situations, providing reasons to recommend explicit policies, training, and oversight rather than relying on clinicians’ individual judgment ( Tamim et al., 2010). Other Saudi-origin research on workplace ethics and unethical behaviours in the hospital setting also underscores the importance of stronger ethics education and institutional governance practices ( Fayez et al., 2013). The data do not suggest that psychotherapy is problematic in a unique way; on the contrary, they indicate that there is a range of ethical interpretations, and the variability itself is a risk factor, particularly when clients are at risk and power disparities are elevated.
5) **An amplifier that is a tight-knit community: social overlap and dual relationship.**
  Another amplifier often used in most Saudi communities is social proximity: clinicians meet clients through family, work connections, communal events, or the Internet. The risk of the dual relationship is thus of a more structural nature, and avoidance may not be feasible. This makes a graduated decision framework more desirable: singling out the cases when it can be avoided, when it cannot be, but can be mitigated with precautions, and when it is disqualifying by virtue of being predictively threatening to the exploitation risk or clinical objectivity ( American Psychological Association, 2017; Gottlieb & Younggren, 2009). Net effect: the combination of these interacting factors, which are moral interpretive environment, regulatory accountability, digitisation, empirical variability, and social overlap, renders a Saudi-adapted, operational boundary framework non-redundant and publishable.

**The contribution of this paper is only secondary data.**

The contribution of this paper is both practical and integrative. It can be undertaken without any ethics committee approvals, since it does not involve any recruitment or data gathering within the framework of this paper. It will combine (i) psychotherapy ethics and research into boundaries, (ii) Saudi legal/professional governance norms, and (iii) Sharia-informed ethical reasoning into one operational package:

1. A Saudi adapted Psychotherapy Boundaries Code of Conduct in categories of risk areas (dual relationships, gifts, mixed gender protection, financial interplay, online interaction, online exposure, and dependency risk).
2. Recurrence dilemma decision rule (e.g., gifts, requests of personal contact, after-hours messaging, family involvement, treating an acquaintance, and tele-therapy as an always-on situation) based on a steady triad: client welfare > risk of exploitation > documentation/consultation ( Gutheil & Gabbard, 1993; Smith & Fitzpatrick, 1995).
3. Recommendations of the institutional governance that perceives boundaries as a system operate: training, supervision pathways, documentation templates, auditfriendly communication policies, and triggers for high-risk situations ( Norris et al., 2007; Tamim et al., 2010).

Such an integrative structure enhances the literature by translating broad ethics into a Saudilegible operational toolkit that can be applied in hospitals, clinics, and university counselling services, without requiring additional data collection.

### Research questions

1. What does the literature of psychotherapy ethics say about crossings and violations of boundaries, and how can harms be produced and maintained ( Hook & Devereux, 2018; Gutheil & Gabbard, 1993)?
2. What are the Sharia’s most relevant principles that affect professional boundary practice (amānah, protection of the dignity, avoidance of risky paths, prohibition of exploitation), and how may they be applied to operational protection ( Kamali, 2003; Padela and Rodriguez del Pozo, 2011)?
3. Which Saudi professional and legal requirements are most applicable to the bounds of obligations when dealing with clinical psychology and allied mental health environments ( SCFHS, 2014; Kingdom of Saudi Arabia, 2018; SDAIA, 2023)?
4. Which, combined, executable “Code of Conduct and decision framework will be able to guide clinicians and institutions that utilise secondary sources alone ( Whittemore & Knafl, 2005)?

## 2. Method (Secondary-data design)

### 2.1 Design overview

This is a secondary-data integrative doctrinal-normative-evidence synthesis paper. It involves no participant recruitment, no surveys, no interviews, and no clinical vignettes. It is a methodologically appropriate fit to integrative review logic, which should be used if the aim is to synthesise the empirical and non-empirical sources (e.g., laws, ethics codes, conceptual scholarship) into an applied system ( Whittemore & Knafl, 2005). It is not to calculate a single pooled effect size, but to generate an operationally useful model that is ethically coherent, legally mindful, and clinically defensible.

Because the field of boundary ethics involves various types of knowledge, a more limited systematic review model would be an inappropriate methodological approach: numerous most appropriate sources are normative (ethics codes), doctrinal (laws), or conceptual (riskmanagement frameworks). Rather, the paper will employ a transparent synthesis approach: the types of sources will be mentioned, as will the selection priorities and integration rules, to allow readers to assess rigour and replicability ( Bowen, 2009; Chynoweth, 2008).

### 2.2 Doctrinal and policy analysis (Saudi sources)

The doctrinal part draws primarily on Saudi laws, regulations, and professional ethics standards. In the logic of doctrinal legal research, it is intended to identify, interpret, and systematise norms relevant to the boundaries that regulate professional practice, misuse of office, conflict of interest, harassment/coercion, conduct related to privacy, and the institution’s responsibility ( Chynoweth, 2008).

Corpus inclusion (document types):

• Ethics standards that govern the practice of specialists in the field of healthcare in the country (e.g., SCFHS ethics instruments) ( SCFHS, 2014).
• Texts of Saudi health practice governance that apply in the field of professional conduct and institutional compliance ( Ministry of Health, 2005).
• Boundary-relevant laws, such as the Saudi Anti-Harassment Law, should be used as a legal measure to address coercive/sexualised misconduct ( Kingdom of Saudi Arabia, 2018).
• Data protection regulation of personal data related to digital jurisdiction and the work with sensitive health-related data ( SDAIA, 2023).

Extraction and coding. Documents are processed using a systematic extractor template that records (a) scope of duty, (b) prohibited behaviours, (c) risk triggers, (d) required safeguards, (e) reporting/escalation pathways and (f ) institutional responsibilities. Subsequently, extracted norms are coded into the following categories relevant to boundaries: dual relationships, gift/benefit conflicts, sexualised conduct/harassment, coercive dependency, privacy-related misconduct (e.g., improper sharing/contact), and digital communication practices ( Bowen, 2009).

The norm map is the output of the doctrinal work: a unified representation of applicable regulations and their relationships to common psychotherapy boundary dilemmas (e.g., gifts, a personal message, economic involvement, or role conflicts).

### 2.3 Normative Sharia analysis

The Sharia commentary is given in the form of normative scholarly thinking (not fatwa issuance). It relies on well-known moral principles, namely, amahnah (trust), safeguarding of dignity, protection against harm, and the avoidance of risky routes, and employs the tools of reasoning found in usul, as well as juristic maxims, in arranging boundary protections ( Kamali, 2003). Where applicable, it appeals to Quranic teachings on trust and the guidance of hadith, not as naive proof-texts, but as valuable policy-level translations of ethics into clinical protection ( Quran.com, n.d.; Al-Tirmidhi, n.d.; Al-Nawawi, n.d.).

The Sharia output is a set of operational restrictions: role clarity, transparency, minimal ambiguity in personal contact, avoidance of benefit extraction, and proactive features of exploitative relationships.

### 2.4 Clinical evidence synthesis

The clinical synthesis prioritises peer-reviewed literature that clarifies:

• definitions and processes (boundary crossings vs violations),
• harms and service-trust damage to the patient,
• risk factors and patterns of the slippery slope, and
• best practice prevention instruments (supervision, documentation, consultation, and institutional policy).

The conceptual risk-management classics ( Gutheil & Gabbard, 1993; Norris et al., 2007), definitional and ethics frameworks ( Smith & Fitzpatrick, 1995; Gottlieb & Younggren, 2009), and patient-harm syntheses ( Hook & Devereux, 2018) are considered high-value evidence. In a culturally embedded dilemma (gifts, gratitude norms), the synthesis involves using literature on how to avoid excessive influence and dependency arising from gifts. In the case of digital boundaries, it applies telepsychology standards and telepsychology scholarship boundary-specific ( JTF, 2013; Drum & Littleton, 2014).

### 2.5 Integration strategy

The process of integration continues in terms of three constraints:

• Legal/professional limitation: the behaviour should be consistent with Saudi Arabian law and professional ethics norms ( Kingdom of Saudi Arabia, 2018; SCFHS, 2014; SDAIA, 2023).
• Sharia necessity and harm-prevention constraint: do not impose morally risky/exploitative terms; do not harm dignity, do not harm foreseeably by role transparency and clarity ( Kamali, 2003; Padela and Rodriguez del Pozo, 2011).
• Clinical proportionality constraint: boundary decisions in the case of the least restrictive option are to be made in a way that they maintain therapeutic integrity with minimal risk of exploitation ( Gutheil & Gabbard, 1993; Gottlieb & Younggren, 2009). The default mechanism when sources are ambiguous is to fall back on conservative protection mechanisms: consult supervision, document the rationale, have little personal contact, and use repeated exceptions as an indicator of boundary drift ( Norris et al., 2007).

## 3. Clinical foundations: Why boundaries matter

### 3.1 Boundaries as a safety structure in an asymmetric relationship

Psychotherapy is asymmetrical in structure: the clinician possesses privileged knowledge and determines the terms of treatment, and they are in the role of an authority, whereas the client might be agitated, dependent, humiliated, or seeking approval. Such asymmetry makes boundaries fundamental, as they ensure that the clinician’s aspirations (validation, emotional rescue, financial benefit, intimacy, status) do not override the client’s ( Gutheil & Gabbard, 1993). The fiduciary relations in which even consent may be impaired when transference, dependency, fear of abandonment, or idealisation of the client influences their ability to refuse.

Clinical judgment is also stabilised through boundaries. When the relationship is of a friendship nature, the therapists can escape needed confrontation, extend therapy to preserve the closeness, or give special exemptions which are caring but end up developing dependence. In the long term, this may undermine treatment objectives, distort alliance reality, and reduce the quality of the outcome, without a dramatic violation event.

### 3.2 Boundary crossings vs violations: a practical distinction

The distinction between bound/violation is irreplaceable, given that psychotherapy is practised in a cross-cultural, cross-institutional setting. The departures from standard parameters are called crossings, which may be clinically reasonable and even advantageous in uncommon situations (such as culturally normative token gifts upon termination or a short message to facilitate stabilisation during a crisis). The moral dilemma is not that the act is out of the ordinary, but that it poses a greater risk of exploitation, violates the roles of a clinical worker, or places covert burdens on them.

In contrast, boundary violations take advantage of the client, indulge the clinician, or harm the client and the treatment in a predictable fashion (e.g. sexual involvement, financial involvement, humiliation, threats and coercive dependency) ( Gutheil & Gabbard, 1993; Smith & Fitzpatrick, 1995). A crossing is a violation if it is repetitive, hidden, clinically unwarranted, self-interested, or if it removes the centre of gravity of the relationship from the client’s therapeutic objectives.

### 3.3 Harm and patient experience: why violations are high-impact ethical failures

The fact that psychotherapy puts people in a vulnerable state under the guise of protection is what causes the harm to be severe and long-term. An analysis of the stories of patients about violation of boundaries reveals the following outcomes: betrayal, shame, reactivation of trauma, fear of seeking help in the future, and long-term distrust of mental health care ( Hook & Devereux, 2018). The damage is also not purely intrapsychic; it may extend to the social and institutional spheres: clients can spread mistrust to the broader system, deterring other clients and relatives from seeking medical attention.

This kind of harm at the system level is particularly applicable in situations where helpseeking is already stigmatised. Boundary governance is part of the public trust and service legitimacy, as even a single scandal can have widespread reputational impacts.

### 3.4 Risk management and the “slippery slope” problem

The academic study of ethics emphasises that most serious breaches start as small rationalisations: additional time, private interaction, confidential contact, exceptions in the name of compassion, or special deals that slowly become normalised ( Gottlieb & Younggren, 2009). The emphasis of risk management is on visibility and structure: it is necessary to monitor when there is a dilemma, record exceptions, and implement institutional policies that reduce clinicians’ isolation ( Norris et al., 2007).

Pattern recognition is a helpful operation indicator: an exception can be tolerated; exceptions that recur, particularly those that lead to more secrecy, more intimacy, more dependency, are indicative of high risk and must prompt consultation. That is not the assumption of ill intent but an evidence-based protection against the expected deviation in highly emotional situations.

## 4. Sharia foundations for professional boundaries

### 4.1 Amānah (trust), dignity protection, and avoiding morally risky pathways

One duty of Sharia ethics is amah: to fulfil trusted obligations with integrity and not turn them into self-interest ( Kamali, 2003). The therapeutic role can be interpreted as amahah since the client delegates personal distress and depends on professional custody as opposed to personal attachment. Such framing is favoured in a default stance of positionality and cautious distancing — not austerity, but disciplined pity.

Sharia also focuses on upholding dignity and discouraging humiliation, exploitation, and unwarranted intrusion into personal affairs ( Chamsi-Pasha & Albar, 2016; Daar & Khitamy, 2001). That is the case in boundary ethics, not just in avoiding unethical actions, but also in avoiding situations that are likely to cause ambiguity, rumour risk, or apparent impropriety, particularly when clients are vulnerable or when environments increase interpretive risk.

### 4.2 *Khalwah/ikhtilāṭ* and professional safeguards: from moral concern to operational design

The prophetic advice that frequently appears in Islamic ethics is that it is dangerous to be secluded between unrelated men and women (khalwah) as a precaution to avoid risks ( AlTirmidhi, n.d.). This means that safeguarding in clinical settings is not aimed at crippling lawful care but creating measures to minimise uncertainty and safeguard clients and clinicians.

This can operationally comprise:

• clinic plans that do not promote ambiguous seclusion (e.g. the use of visibility panels indoors where it is necessary to use them),
• express appointment and chaperone policies in particular circumstances (particularly in the presence of minors),
• clear records of time and place of session, and
• professional communication not based on any personal relational indicators ( Padela and Rodriguez del Pozo, 2011).

Notably, these measures can be presented as dignity-protective rather than suspicion-driven: they help clients feel safe and minimise the risk of therapeutic intimacy being misunderstood as personal intimacy.

### 4.3 Gifts, gratitude, and avoiding undue influence

The morality of Sharia also contains many warnings against gift-giving as leverage or to involve a person in obligations, particularly those in a position of responsibility ( Al-Nawawi, n.d.). Although psychotherapy does not have the same ethical code as the office of the people, the same ethical mechanism can be involved: the gifts may produce an undue influence, nurture the dependency, or distort the therapeutic neutrality.

The meaning, timing, and value of gifts and transference/countertransference are also discussed in the literature on clinical ethics. A culturally sensitive approach does not mean refusing all gift tokens, though structures fundamentally required: nominal value, transparency, documentation, avoiding secrecy and giving gifts more of a therapeutic (exploring meaning) than a relationship (reciprocity) approach.

### 4.4 Exploitation ( *istighlāl al-tabʿiyyah*) and the prohibition of taking advantage of dependency

One of the key Sharia issues is the exploitation of vulnerability: the ability to use power to receive a personal gain at the expense of a person who is at a disadvantage or cannot say the word and refuses ( Kamali, 2003). The client’s consent in psychotherapy is ethically insecure due to the possibility of dependency and idealisation included in the clinical image. This is the reason why sexual liaisons, financial conflicts and manipulative emotional bondage are considered as exploitation violations in the professional ethics codes ( American Psychological Association, 2017; Gutheil & Gabbard, 1993).

Such an intersection is important to Saudi integration: both Sharia-informed ethics and mainstream psychotherapy ethics use exploitation risk as a boundary marker. Hence, not only can the imposed prohibitions be defended by the integrated framework as imported professional norms, but also as locally sounding dignity-protective responsibilities.

## 5. Saudi regulatory and professional landscape

### 5.1 Professional ethics codes and conduct expectations

The standards of Saudi professional ethics provide requirements of integrity, respect, and professionalism on the part of healthcare practitioners. The SCFHS code of ethics serves as a primary normative reference point for role-based behaviour and patient protection, promoting the incompatibility of misconduct and exploitation with professional identity ( SCFHS, 2014). In practice, this facilitates institutional training needs, lines of complaint, and disciplinary procedures, which are mechanisms that aid in bringing ethics to everyday practice.

### 5.2 Anti-harassment protections and boundary-relevant prohibitions

Saudi Anti-Harassment Law enhances the boundary framework by establishing and prohibiting harassment practices and legal intolerance of coercive or sexualised behaviour ( Kingdom of Saudi Arabia, 2018). Legal reinforcement issues: even in situations where psychotherapy ethics would already preclude such behaviour, the presence of legal guarantees of such behaviour helps make it more deterrence-enhancing, enables reporting, and facilitates institutional escalation. In the case of boundary policy, it would mean that psychotherapy boundary policies must be clearly linked to broader legal safeguards against coercion and harassment, particularly where there is a power imbalance.

### 5.3 Healthcare practice regulation and institutional accountability

The Saudi health practice governance books serve as the foundation for legal, ethical, and professional practice expectations ( Ministry of Health, 2005). The most important practical implication in the case of boundary ethics is that institutions should view boundaries as professional compliance: documentation, regulations, role definitions, conflict avoidance, and accountability mechanisms are not mere add-ons; they are fundamental components of the safe and legal provision of healthcare.

### 5.4 Data protection and digital boundary compliance

Privacy compliance cannot be separated from digital boundaries. The governance of Saudi personal data protection aligns with expectations of minimisation, purpose restriction, data security, and limited access to data, which overlap directly with clinical messaging, record management, and telecare practices ( SDAIA, 2023). In clinical practice, digital informality may lead to role confusion and data exposure: late-night messaging, personal device use, unrecorded advice, or contact with social media may result in dependency processes and also provide an audit trail of inappropriate practice.

Informed consent, privacy planning, documentation, and emergency pathways are the key aspects of remote care that Telepsychology guidance focuses on ( JTF, 2013). Boundaryspecific telepsychology scholarship also cautions that remote environments are prone to hastening boundary drift due to confusion over time/place norms and the convenience of contact ( Drum & Littleton, 2014). Thus, Saudi-adapted Boundary governance must include specific Saudi-appropriate measures, namely: digital professionalism regulations; standard channels, response time, and documentation; and harsh prohibitions on social media entanglement or personal relationship indications.

## 6. Core dilemmas and decision rules (Secondary-data synthesis)

This part of the paper renders the integrative logic into repeatable decision rules to apply whenever faced with everyday boundary dilemmas. This is not aimed at producing a hardand-fast one-size-fits-all checklist, but a defensible professional reasoning pathway that is (a) clinically protective to the client, (b) compatible with Sharia-informed moral expectations (am 2nah, dignity, prevention of morally risky pathways), and (c) compliant with Saudi professional governance and accountability standards ( APA, 2017; SCFHS, 2014; Chamsi-Pasha & Albar, 2016).

Two conceptual anchors control the synthesis:

• Fiduciary asymmetry: psychotherapy is organizationally unequal, in terms of power, privileged information, and control, with it may be in clinicians, who are authorities, possess privileged information, and control parameters; or in clients, who are distressed, dependent, or seeking approval. Boundaries minimise the threat that the client’s welfare will be displaced by the therapist’s needs ( Gutheil & Gabbard, 1993; Smith & Fitzpatrick, 1995).
• The fallacy of the slippery slope: It is easy to start as a minor exemption (additional contact, unstructured favours, secrecy, or special status). The prevention model thus places greater emphasis on visibility, consultation, documentation, and institutional protection than on the virtue of an individual ( Gottlieb & Younggren, 2009; Norris et al., 2007).

### 6.1 Mixed-gender sessions, privacy, and khalwah-sensitive risk management

Why is it of clinical and normative significance? Mixed-gender care has become standard practice in contemporary care, and a blanket ban would be detrimental to patients and access. However, in a moral economy of Sharia-informed settings, the manner in which the session is set can have ethical and reputational significance both to the client and the clinician, particularly in situations where the stigma of social forces is high, family matters and communal invisibility are high ( Padela & Rodriguez del Pozo, 2011; Daar & Khitamy, 2001). In a clinical perspective, it is not a matter of gender rules but of risk management: to minimise unnecessary ambiguity, avoid claims, preserve dignity, and maintain patient comfort, allowing the therapeutic alliance to develop ( Gutheil & Gabbard, 1993; Hook & Devereux, 2018).

Decision rule (integrated).

• Clinical legitimacy first: in no case should morbid care be hampered by excessive fear; legitimate treatment is morally and professionally good.
• Protection is needed when the situation is more vulnerable (e.g. minors, conflictual family life, high profile, or prior accusations).
• Select the least invasive protection that minimises the ethical risks of isolation and ambiguity, preserves privacy, and maintains treatment efficacy ( Gottlieb & Younggren, 2009).

Protection of operations (practice-facing).

1. Professional visibility, but not public exposure. Visibility refers to avoiding anonymity, which can foster suspicion, rather than making therapy an observable phenomenon. The design alternatives for the clinic include: a door window with privacy film, a room location with controlled access to corridors, or an institutional door policy that is uniform and written ( Padela & Rodriguez del Pozo, 2011). This is to minimise ambiguity and maintain confidentiality.
2. Location discipline. Do not hold informal and private meetings outside of clinical environments (homes, cafes, cars). Even if the intentions are benign, the setting itself might cause confusion about boundaries, lead to accusations, and undermine dignity ( Gutheil & Gabbard, 1993).
3. Systematised logistics records. Record the date/time/location, start and end times, and institutional setting as a standard precautionary practice in sensitive settings ( Norris et al., 2007). This is not the suspicion of documentation; it is government.
4. Chaperone/observer in case of clinical necessity. This must be presented as a clientprotection choice, not a default intrusion. It is particularly applicable to minors, highrisk family conflicts, or situations in which institutional policy requires the protection of structures ( Norris et al., 2007).
5. Touch discipline and proximity discipline. Physical contact in psychotherapy can hardly be essential and can even be clinically charged. In cases where safety requires physical support (e.g., a medical emergency), it must be kept to a minimum and documented. This minimises the moral risk and post-hoc misinterpretation ( Gutheil & Gabbard, 1993).

Ethical justification (Sharia clinical synthesis). Sharia-informed ethics focuses on amānah (role integrity) and avoiding morally hazardous courses, whereas clinical ethics focuses on an individual’s vulnerability to exploitation and on avoiding coercive ambiguity ( Chamsi-Pasha & Albar, 2016; Gutheil & Gabbard, 1993). A safeguards-based model operationalises both: it safeguards dignity, eliminates the risk of allegations, and maintains access to care. In addition to internationally recognized psychotherapy ethics frameworks ( American Psychological Association, 2017), counseling ethics guidance emphasizes the prevention of harm through clear role boundaries, avoidance of dual relationships, and management of conflicts of interest ( American Counseling Association, 2014), while Islamic medical ethics literature highlights trust (amānah), dignity protection, and professional integrity as core duties in clinical relationships ( Chamsi-Pasha & Albar, 2016).

### 6.2 Gifts and hospitality

The reason why gifts are not simple (ethical). Gifts may be normative expressions of thanks in collectivist cultures; however, clinically, they may also be vectors of transference, dependency, and obligation. The therapist should thus be able to assess the monetary value, the relational value of the item and also its potential effects on treatment limits ( Smith & Fitzpatrick, 1995). A gift may (a) cement alliance openly and humbly, (b) develop expectations of favouritism, privacy and incremental confusion.

Decision rule.

• Not only value, but assess meaning + risk.
• Revert to polite refusal except when this refusal would result in disrupting the culture or in producing clinical harm.
• Acceptance should be made only with clinical justification and only for nominal items; this approach should be done transparently, documented, and receive therapeutic processing ( Gottlieb & Younggren, 2009).

Considerable assessment criteria (synthesis of secondary data).

1. Timing: Gifts given in the midst of intense dependency, crisis, or idealisation carry a greater risk than those given at termination.
2. Value/scale: high values of gifts enhance leverage and pressure on the other side; nominal gifts can be controlled in case they are recorded and reported.
3. Intent/meaning: Gratitude is distinguished by leverage (I expect special access), repair attempt (Do not abandon me), or control (You owe me).
4. Secrecy: any demand of secrecy (Do not tell anyone I gave you this) is a significant alert to the boundary erosion ( Gottlieb & Younggren, 2009).
5. Consequences: Are there any predictable consequences of acceptance of out-ofsession demands, entitlement, or inequality among clients?

Preference (clinically realistic).

• Refuse to use a script that is respectful and does not give up dignity: “What you appreciate is important; what you are doing is most important; I have gifts there so that we stay focused on you at work.
• In case it is appropriate to say yes: accept small, culturally normative objects; write down; and briefly discuss meaning in the session (What does giving this mean to you?). This introduces a potential risk of crossing a boundary at a clinically useful moment while preserving boundaries.
• Accept none of the gifts that produce financial dependence, expectation repetition, and business opportunities.

Sharia–clinical synthesis. The Sharia morals discourage presents that serve as forms of undue influence in relationships of authority, which support the notion that the position should not be bought and that the vulnerable should not be exploited ( Daar & al Khitamy, 2001; ChamsiPasha & Albar, 2016). Clinical ethics meets: even small gifts can be stepping stones in a chain of slippage at the border, in case secrecy and special status become normal ( Gottlieb & Younggren, 2009).

### 6.3 Conflicts of interest and dual relationships

The reason why a dual relationship is structurally hazardous. The dual relationship (two roles with the same individual, therapist + teacher, etc.) is the one that logically threatens objectivity and makes the therapist more likely to exploit the person, since their motivations are split. The therapist may also be motivated by the desire to defend the other relationship rather than confront clinically necessary issues (or the client may be intimidated by the social costs of adhering to therapy) ( APA, 2017; Gutheil & Gabbard, 1993).

Decision rule.

• A dual role should be avoided whenever possible.
• In cases where it is unavoidable (e.g., small communities with limited providers), use strong precautions: clear consent, role definition, supervision/consultation, documentation, and boundaries on information flow ( APA, 2017; Gottlieb & Younggren, 2009).

Ordinary Saudi-salient dual-role situations.

1. Supervisees, trainees and students. The asymmetry of power is increased by a factor of 2 (academic + clinical), and the risk of coercion is increased.
2. The institutional and the workplace overlap. Generosity towards colleagues, subordinates, or people within one governance structure heightens reputational and conflict risks.
3. Tight-knit communities. The proximity of the social group increases accidental encounters and role drift; thus, the policy should not ignore this fact but plan for it ( Gottlieb & Younggren, 2009).

Safeguard package (minimum standard in case dual roles are inevitable).

• Role-limits consent: plain language account in writing that therapy is not going to be used in grading, promoting, business entertaining or family arbitration.
• Information firewall: different channels and few disclosure rules; do not talk casually out of session.
• Consultation requirement: seek supervision or peer consultation early, not when problems arise ( Norris et al., 2007).
• Referral priority: if the referral is possible at a later time, re-evaluate and relocate to protect the client’s autonomy and eliminate the risk of coercion ( APA, 2017).
• Documentation: rationale and safeguards of records; documentation distinguishes between a controlled exception and rationalised drift.

### 6.4 Exploitation of dependency and sexual boundary prohibitions

Non-negotiable principle. Sexual relationships with existing therapy clients fall under fiduciary care as relationships that are structurally incompatible since there is no apparent consent between them and the therapy client due to dependence, transference, and authority of the therapist. That is why it is a serious violation, as prescribed by professional codes, rather than a relationship choice ( APA, 2017; Gutheil & Gabbard, 1993).

Wider oppression other than sexuality. The boundary framework provided by a contemporary Saudi with Saudi-specific modifications should also clearly deal with non-sexual exploitation: financial exploitation, favours, loyalty, social status based on therapy, or forcing dependence (Only I can help you). These actions are clinically disastrous, as they transform therapy into a process of control and shaming ( Hook & Devereux, 2018; Norris et al., 2007). Long-term care: post-termination relations. Relational power residue may be present even after being dismissed — particularly in the case of a client with a history of trauma, attachment wounds, or idealisation. Scholarship in the ethics field has noted that risk does not disappear during termination; instead, termination may be used to rationalise (We waited until therapy ended), yet dependency persists ( Gutheil & Gabbard, 1993; Smith & Fitzpatrick, 1995). The justifiable one is the high level of caution, consultation, and avoidance in case of any attraction or benefit motive ( APA, 2017).

Prevention measures (what a good clinic anticipates).

• Attraction management procedure. There may arise feelings of attraction, and the violation in this case is an action. A clinic ought to normalise consultation (Should attraction arise, consult and record what was done to safeguard the client) ( Norris et al., 2007).
• Discipline of the structure of the session. Time, location, and work-related professionalism make the transition to the intimacy roles predictable.
• No secrecy norm. Crimes flourish in solitude, and, as such, institutions have to reduce therapists’ isolation through a supervision culture ( Norris et al., 2007).
• Boundary clarification at an early stage. Abandonment fears can be tested in clients. Another boundary stance, clear and caring on the part of the counsellor, does not lead to escalation.

### 6.5 Social media, messaging apps, and tele-mental health boundaries

The reason why digital contact is a boundary accelerant. Digital care alters the default settings of care: it becomes easier to stay in contact, more frequent and less formal; it lacks time sources; and it enhances the longevity of the records (screenshots, forwards). This might create clinical dependency (presented by the availability of a therapist at all times) and privacy risk (information loss, sharing devices) ( JTF, 2013; Drum & Littleton, 2014). What it means is that digital boundaries must be treated as central to professionalism, rather than as a personal style preference.

Decision rule.

• Establish and implement a policy for digital communication specifying Authorised channels, response times, content restrictions, emergency routes, and documentation ( JTF, 2013; Drum & Littleton, 2014).
• Use institution-approved services and devices for tele-mental health, and avoid saving clinical material on personal devices whenever possible.
• Do not friend/follow current clients on social media due to the risk of dual relationships and breaches of confidentiality ( APA, 2017).

Effective policy factors (minimum).

1. Channels: define which platform to use for scheduling vs therapy content. Low-risk scheduling is possible; the content of therapy must be secured and recorded.
2. Time: establish response times and anticipated delay (e.g. responses within 24–48 hours (during working days). This helps avoid unstructured dependence and reduces therapists’ burnout, thereby lowering the risk of boundary erosion ( Drum & Littleton, 2014; Gottlieb & Younggren, 2009).
3. Content: forbid crisis management in plain text; establish the emergency route (emergency services, hospital, hotline, on-call clinic).
4. Documentation: clinical record summarisation should include out-of-session communication that is clinically meaningful ( JTF, 2013).
5. Privacy education: the clients must be trained to be less visible at home (shared devices, notifications, and family members overhearing). Tele-therapy privacy is cocreated; the informed consent needs to be specific to this issue ( JTF, 2013). Saudi governance overlay. The Saudi environment of personal data protection is also involved in the digital boundaries. Health and mental health data are among the most sensitive; they should be minimally exposed, access to them should be limited, and secure processing practices must be employed ( SDAIA, 2023). Consequently, informal messaging to deliver clinical content via WhatsApp is not just a boundary problem but, in fact, a datagovernance problem.

## 7. Proposed Saudi “Code of Conduct” for Clinical Psychologists

The code proposed is intended for institutional implementation as a governance instrument, not as a moral declaration. It is meant to normalise, reduce vagueness, and assist with standard supervision and record keeping. It aligns with the reasoning of contemporary codes of ethics ( APA, 2017) and is culturally sensitive to the Sharia-based moral expectations of dignity and trust ( Chamsi-Pasha & Albar, 2016; Alotaibi, H. A., 2020). It is also indicative of the Saudi professional ethics environment ( SCFHS, 2014).

### 7.1 Preamble: Boundaries as patient-safety infrastructure

Ethics safeguard the clients and clinicians. They maintain the therapeutic bond as a fiduciary zone in which therapeutic application may be objective, the client’s autonomy is honoured, and vulnerability is not used to the therapist’s advantage ( Gutheil & Gabbard, 1993). Boundary policy should be considered a quality and safety issue, not an optional ethics addon by institutions ( Norris et al., 2007).

### 7.2 Core principles (with operational meaning)

**Principle 1: Fiduciary Integrity (Amah)**

Treatment objectives define the clinician’s role. 1. Any form of action is outlawed, and the main aim is to attract a clinician’s emotional, social, sexual, or financial interests. Institutional implication: the decision to regard the boundaries should be justified regarding client welfare ( Chamsi-Pasha & Albar, 2016; APA, 2017).

**Principle 2: Non-Exploitation and Dignity**

Guard the client against humiliation, coercion and dependency exploitation. Institutional implication: establish transparent anti-exploitation regulations and avenues of safe reporting ( Hook & Devereux, 2018; Norris et al., 2007).

**Principle 3: Default Clarity of Boundaries**

Have a consistent session format, roles, and communication lines. The exceptions are minor and have to be explained and recorded. This will minimise the rationalisation route to violations ( Gottlieb & Younggren, 2009).

**Principle 4: Proportionality and Minimalist Alternative**

When considering a boundary crossing, select the least deviation that would be clinically beneficial and reduce role confusion ( Gottlieb & Younggren, 2009).

**Principle 5: Transparency, Consultation and Documentation**

The boundary dilemma creates a need for supervision/peer consultation rather than individual discretion. Material boundary events, dual roles, and significant out-of-session contact must be documented ( Norris et al., 2007).

**Principle 6: Digital Professionalism and Protection of Data**

The digital contact should be designed to be safe and aligned with informed consent and privacy governance ( JTF, 2013; SDAIA, 2023).

### 7.3 “Red lines” (non-negotiables)

• The sexual or romantic involvement with existing clients ( APA, 2017; Gutheil & Gabbard, 1993).
• Financial involvement (loans, business associations, referral fees).
• The use of coercion, threats, humiliation, retaliation or exploitative dependency.
• Undisclosed contact, which is non-institutional.
• There is unsecured processing of sensitive patient data and unofficial sharing that can expose them to risks ( SDAIA, 2023).

### 7.4 Documentation requirements (minimal template logic)

Institutions are required to provide a short formal note where:

• gift offered/accepted/refused;
• a dual relationship is present or inescapable;
• There is an exemption to the digital communication policy.
• The client or staff initiates a boundary issue.

This makes transparency the order of the day and less secrecy, which is the best indicator of boundary problem escalation ( Norris et al., 2007; Gottlieb & Younggren, 2009).

## 8. Implementation: Training, supervision, and governance

A boundary framework cannot work when it is treated as personal morality. Systems are needed to prevent: training, supervision, documentation templates and governance pathways. According to the literature on boundary problems, consultation and education by career stage are fundamental prevention measures ( Norris et al., 2007). It can be applied in the same vein in Saudi clinical units, particularly given the rapid pace of digitisation and the heightened sensitivity of the population to professional malpractice.

### 8.1 Training modules (secondary data, policy-scenario based)

Policy scenarios may be used in training without gathering research information or using vignettes as a study design. Modules should include:

• The principles of fiduciary limits: power asymmetry, transference/countertransference and the slippery-slope pattern ( Gutheil & Gabbard, 1993; Gottlieb & Younggren, 2009).
• Gifts and hospitality: cultural sensitivity, decision factors and documentation requirements.
• The issue of dual relationships in close communities: risk assessment, consultation provocation, and disciplining decision referral ( APA, 2017).
• Tele-mental health, informed consent updates, secure channel, response window, and recordkeeping ( JTF, 2013; Drum & Littleton, 2014).
• Informed professionalism under Sharia: amānah, dignity, and avoidance of controversially risky courses of action as a blessing ( Chamsi-Pasha & Albar, 2016; Padela and Rodriguez del Pozo, 2011).
• Saudi professional accountability overlay: dependence on the national ethics expectations and institutional policies ( SCFHS, 2014).

### 8.2 Supervision and consultation pathways

Boundary dilemmas are supposed to trigger compulsive consultation rather than be left to lone decision-making. Consultation minimises rationalisation and promotes the existence of consistent institutional standards and defence documentation ( Norris et al., 2007). A practical model includes:

• a bill called ethics/safeguarding lead on queries concerning boundaries;
• a fast-track guidance system on emergency cases;
• a second-opinion condition of dual relationships or high-stakes digital exemptions.

### 8.3 Documentation templates (make the “right action” easy)

The templates should be brief enough to be used regularly. They should record:

• What happened (description of events),
• ethical risk factors,
• rule of decision used (e.g. refusal/acceptance with reason),
• consultation obtained,
• client communication,
• follow-up plan.

Client and clinician protection are both supported by documentation, a point constantly reiterated in the literature on boundary risks ( Norris et al., 2007).

### 8.4 Institutional policy alignment and audits

The boundary policy should be consistent with institutions:

• SCFHS, 2014 national professional ethics expectations,
• tele-mental health privacy ( JTF, 2013), and
• personal data protection principles (secure processing) (minimisation) ( SDAIA, 2023).

The audits must target the process risks and not just bad actors:

• record access logs;
• use of an inappropriate channel of messaging;
• gifts/dual roles documentation;
• compliance with the policy of the response window;
• knowledge of the paths of escalation among the staff.

### 8.5 Safe reporting and non-retaliation culture

Fear, shame, or reputational pressure leads to underreporting of boundary violations. Organisations must introduce confidential reporting mechanisms and implement nonretaliation provisions through leadership communication, presenting reporting as patient safety rather than crisis management ( Hook & Devereux, 2018; Norris et al., 2007).

## 9. Conclusion

Professional boundaries are neither a nicety nor an etiquette; they are a fiduciary, powerasymmetric relationship, an architectural structure of therapeutic safety. Clinical sources include much more emphasis on the fact that the violation can cause significant damage, such as betrayal trauma, mistrust, and avoidance of mental health care in the long term ( Hook & Devereux, 2018). Scholarly work on ethics also demonstrates that many violations occur through rationalised exceptions; thus, preventing such violations is a systems issue that requires consultation, documentation, and design ( Gottlieb & Younggren, 2009; Norris et al., 2007).

Moral expectations based on Sharia convergence which focuses on amānah, dignity, and avoidance of morally risky pathways; professional governance structures that require integrity and shun exploitative behavior; and digital transformation which intensifies boundary drift due to ubiquitous messaging, tele-therapy, and increased data exposure amplify the boundary questions in the Saudi context ( Chamsi-Pasha & Albar, 2016; JTF, 2013; SDAIA, 2023). These forces do not abstractly complicate ethical practice; they render clarity and governance more significant.

In this way, the contribution of this paper is a secondary-data, integrative framework, which can be adopted without the fieldwork: (1) a decision rule regarding the most widespread boundary dilemmas (mixed-gender protections, gifts, dual roles, exploitation, digital contact), (2) a Code of Conduct framed to suit Saudi culture, and (3) implementation mechanisms (training, supervision, templates, audits) that will turn boundaries into individual discretion into organisational patient-safety infrastructure ( APA, 2017; Norris et al., 2007; SCFHS, 2014).

Lastly, the framework deliberately addresses the concept of boundary ethics as both ethical obligation and service-quality approach: as long as clients have a positive experience of safe and reliable therapy due to the demonstration of an apparently clear and consistent professionalism (particularly regarding gifts, dual roles, and online accessibility), they tend to perceive the mental health services as safer and more trusting and believe in it, which in turn protects the reputation of this type of services in general ( Gutheil & Gabbard, 1993; Drum & Littleton, 2014). This aligns with Vision-related objectives of enhancing community trust in professional services without undermining dignity, privacy, and safety in a rapidly modernising health sector.

## Data Availability

No new participant level datasets were generated or analysed in this study. This article is a secondary-data doctrinal normative evidence synthesis based exclusively on publicly available Saudi legal and professional regulatory texts and published peer-reviewed literature, all cited in the reference list. Available at: https://doi.org/10.6084/m9.figshare.31424660 ( Alotaibi & Alotaibi, 2026). Repository name: Figshare: *Professional Boundaries – Saudi Sharia–Legal–Clinical Framework.* Available at: https://doi.org/10.6084/m9.figshare.31424660 ( Alotaibi & Alotaibi, 2026). The project contains the following underlying data:
•
Source_corpus_register.xlsx (complete register of legal, regulatory, and peer-reviewed sources used in the synthesis, including URLs and DOIs where available). • Extraction_matrix.xlsx (structured mapping of sources to boundary-risk domains and operational principles). • Search_protocol.txt (databases searched, keywords, inclusion/exclusion criteria, and screening method). Repository name: Figshare: *Professional Boundaries – Saudi Sharia–Legal–Clinical Framework.* Available at: https://doi.org/10.6084/m9.figshare.31424660 ( Alotaibi & Alotaibi, 2026). This project contains the following extended data:
•
Operational_toolkit_appendix.pdf (Saudi-tailored Code of Conduct, decision rules, and documentation templates for institutional implementation). This content is licensed as CC BY 4.0.
