# Supplementary material for: Professional Boundaries and Ethical Obligations in Saudi Arabia An Integrated Sharia, Legal and Clinical Psychology Framework
Source: F1000Res. 2026 Jun 1;15:461. Originally published 2026 Apr 1. [Version 2] doi: 10.12688/f1000research.178561.2 (PMC13187715; doi:10.12688/f1000research.178561.2)
Supplement: Peer review history

# Reviewer report: Reviewer response for version 2

**DOI:** 10.5256/f1000research.202002.r489883

**Published:** 2026-06-23

**Authors:** Dwi Fidhayanti

**Affiliations:** 1 UIN Maulana Malik Ibrahim Malang, East Java, Indonesia

I appreciate the detailed review. The revision are clear, and I have no additional comments to add.

Is the work clearly and accurately presented and does it cite the current literature?

Partly

If applicable, is the statistical analysis and its interpretation appropriate?

Not applicable

Are all the source data underlying the results available to ensure full reproducibility?

Partly

Is the study design appropriate and is the work technically sound?

No

Are the conclusions drawn adequately supported by the results?

Yes

Are sufficient details of methods and analysis provided to allow replication by others?

No

Reviewer Expertise:

my research focuses primarily on Islamic Economic Law (Hukum Ekonomi Syariah), with a particular emphasis on sharia compliance and legal certainty within the digital economy, such as Islamic fintech, e-commerce regulations, and consumer data protection. Additionally, I engage in deep socio-legal studies concerning local cultural-legal phenomena in Indonesia.

I confirm that I have read this submission and believe that I have an appropriate level of expertise to confirm that it is of an acceptable scientific standard.

# Reviewer report: Reviewer response for version 2

**DOI:** 10.5256/f1000research.202002.r489881

**Published:** 2026-06-17

**Authors:** Ramadhita Ramadhita

**Affiliations:** 1 Universitas Islam Negeri Maulana Malik Ibrahim Malang, Malang, East Java, Indonesia

The manuscript now presents a more coherent argument, demonstrates a stronger theoretical and methodological foundation, and offers a clearer contribution to the existing body of knowledge. The authors’ efforts to engage constructively with the review comments are evident throughout the revised version.

Is the work clearly and accurately presented and does it cite the current literature?

Yes

If applicable, is the statistical analysis and its interpretation appropriate?

Not applicable

Are all the source data underlying the results available to ensure full reproducibility?

Yes

Is the study design appropriate and is the work technically sound?

Yes

Are the conclusions drawn adequately supported by the results?

Yes

Are sufficient details of methods and analysis provided to allow replication by others?

Yes

Reviewer Expertise:

islamic law; family law; cultural studies

I confirm that I have read this submission and believe that I have an appropriate level of expertise to confirm that it is of an acceptable scientific standard.

# Reviewer report: Reviewer response for version 1

**DOI:** 10.5256/f1000research.196957.r473978

**Published:** 2026-05-19

**Authors:** Sholahuddin Al-Fatih

**Affiliations:** 1 Universitas Muhammadiyah Malang, Malang, Indonesia

The manuscript sounds good. However, the reference looks too old. Please read the newest article from an internationally reputable journal as well as the last 5 years (2020=2025). Then, the authors should elaborate on the conceptual or theoretical framework with the empirical data. Furthermore, it would be like more attractive while the authors also provide the comparative perspective with the related topic, such as in UEA or several Middle East countries

Is the work clearly and accurately presented and does it cite the current literature?

Yes

If applicable, is the statistical analysis and its interpretation appropriate?

Yes

Are all the source data underlying the results available to ensure full reproducibility?

Yes

Is the study design appropriate and is the work technically sound?

Yes

Are the conclusions drawn adequately supported by the results?

Yes

Are sufficient details of methods and analysis provided to allow replication by others?

Yes

Reviewer Expertise:

legal studies

I confirm that I have read this submission and believe that I have an appropriate level of expertise to confirm that it is of an acceptable scientific standard.

## References

[1] : Harmonising Legal and Sharia Principles in Foreign Investment: The Regulatory Framework of Subsidiaries in Saudi Arabia. *Legality : Jurnal Ilmiah Hukum* .2026;34(1) : 10.22219/ljih.v34i1.42145 162-182 10.22219/ljih.v34i1.42145

# Reviewer report: Reviewer response for version 1

**DOI:** 10.5256/f1000research.196957.r483701

**Published:** 2026-05-18

**Authors:** Dwi Fidhayanti

**Affiliations:** 1 UIN Maulana Malik Ibrahim Malang, East Java, Indonesia

The manuscript presents an innovative and highly relevant integrative framework that combines clinical ethics, normative Sharia principles ( *amānah*, dignity, prevention of *khalwah*), and Saudi Arabian regulations (SCFHS, SDAIA, Anti-Harassment Law) to structure professional boundaries as an organizational patient-safety infrastructure. The conclusions drawn in Section 9 are adequately supported by the decision rules and the drafted Code of Conduct developed in the preceding sections, while the statistical analysis criterion is deemed Not Applicable due to the study's qualitative-normative methodology. However, to ensure full reproducibility, the authors **must address two critical points**: first, they need to attach the blank "systematic extractor template" or coding matrix referenced in Section 2.2 as an Appendix; second, they must resolve text anomalies by correcting the typographical error in Section 6 (changing *"am 2nah"* to *"amānah"*) and completing the missing end of Section 8.1, which currently truncates abruptly mid-citation at *"Gutheil & Gabbard"*.

Is the work clearly and accurately presented and does it cite the current literature?

Partly

If applicable, is the statistical analysis and its interpretation appropriate?

Not applicable

Are all the source data underlying the results available to ensure full reproducibility?

Partly

Is the study design appropriate and is the work technically sound?

No

Are the conclusions drawn adequately supported by the results?

Yes

Are sufficient details of methods and analysis provided to allow replication by others?

No

Reviewer Expertise:

my research focuses primarily on Islamic Economic Law (Hukum Ekonomi Syariah), with a particular emphasis on sharia compliance and legal certainty within the digital economy, such as Islamic fintech, e-commerce regulations, and consumer data protection. Additionally, I engage in deep socio-legal studies concerning local cultural-legal phenomena in Indonesia.

I confirm that I have read this submission and believe that I have an appropriate level of expertise to confirm that it is of an acceptable scientific standard, however I have significant reservations, as outlined above.

# Response

**Published:** 2026-05-18

**Authors:** Hajed A. Alotaibi

**Affiliations:** Majmaah University, Saudi Arabia

Thank you for your valuable comments, informative guidance. Here are our resplies to the reviewer 1 comments: A) Reproducibility: blank extractor template/coding matrix

- Response: We appreciate the focus on reproducibility. The blank systematic extractor template/coding matrix is hosted openly in our Figshare project (DOI: 10.6084/m9.figshare.31424660). We cross-linked it in Section 2.2, Data availability, and Extended data. To preserve a single version-controlled source, we did not embed it as an appendix.
- Manuscript insertions: • Section 2.2 (final sentence of “Extraction and coding”): The blank systematic extractor template/coding matrix is openly available in our Figshare repository and cross-linked in Data availability and Extended data; readers can download the exact fillable form used. • Data availability (new bullet): Blank systematic extractor template/coding matrix (fillable): Figshare, DOI: 10.6084/m9.figshare.31424660. This serves as the authoritative reusable template referenced in Section 2.2. • Extended data (new bullet): Blank systematic extractor template/coding matrix (fillable), Figshare, DOI: 10.6084/m9.figshare.31424660 (cross-referenced in Section 2.2 and Data availability).

B) Typographical error “am 2nah” → “amānah”

- Response: Corrected in Section 6 where it first appears.

C) Truncated sentence in Section 8.1

- Response: Completed the line in 8.1 as follows: • The principles of fiduciary limits: power asymmetry, transference/countertransference, and slippery-slope dynamics (Gutheil & Gabbard, 1993; Gottlieb & Younggren, 2009).

# Reviewer report: Reviewer response for version 1

**DOI:** 10.5256/f1000research.196957.r475386

**Published:** 2026-05-18

**Authors:** Ramadhita Ramadhita

**Affiliations:** 1 Universitas Islam Negeri Maulana Malik Ibrahim Malang, Malang, East Java, Indonesia

1. Strengthening the Analytical Contribution (Beyond Synthesis)

 While the manuscript successfully synthesizes three normative domains (Sharia, law, and clinical ethics), its **analytical added value remains somewhat implicit**. At present, the paper tends toward descriptive integration rather than critical interrogation.

- The authors should more explicitly articulate:
  - *What tensions or inconsistencies exist* between Sharia norms, Saudi regulations, and global clinical ethics frameworks?
  - *How does the proposed framework resolve or prioritize* when conflicts arise?
- For instance, in areas such as mixed-gender interaction or digital communication, there may be latent normative friction that is not fully explored but rather harmonized prematurely.

A stronger critical layer would elevate the manuscript from integrative review to **normative-theoretical contribution**.

 2. Methodological Rigor and Justification

 The manuscript adopts a “secondary-data integrative doctrinal-normative-evidence synthesis” design , which is appropriate for the research aim. However, the **methodological execution requires deeper justification and transparency**:

- The integration strategy is described conceptually, but **not sufficiently operationalised**:
  - How are conflicting sources adjudicated?
  - Is there a hierarchy (e.g., legal norms > ethical norms > clinical evidence)?
- The selection of sources appears authoritative but **risks being perceived as selective** without explicit criteria.
- The paper would benefit from a clearer explanation of **how doctrinal legal analysis and normative Sharia reasoning are methodologically combined**, as these are epistemologically distinct traditions.

Without this clarification, the framework may appear normatively persuasive but **methodologically underdetermined**.

 3. Depth of Sharia Engagement

 The use of Sharia principles (e.g., *amānah*, dignity, avoidance of harm, *sadd al-dharā’iʿ*) is appropriate and relevant. However, the engagement remains **relatively high-level and generalised**:

- The manuscript would benefit from:
  - More **explicit usul al-fiqh reasoning pathways** (e.g., qawāʿid fiqhiyyah, maqasid hierarchy, or juristic disagreement where relevant)
  - Clarification on whether the framework reflects a **specific madhhab orientation or a generalised Sunni synthesis**
- Currently, Sharia is presented as largely convergent with clinical ethics. While this is plausible, it risks **oversimplification of internal plurality within Islamic jurisprudence**.

A more nuanced treatment would significantly strengthen the paper’s credibility in Islamic legal scholarship.

 4. Contextualisation within Existing Literature

 The manuscript cites key classical and contemporary works, but its **positioning within current international debates is somewhat limited**:

- The authors should clarify:
  - How this framework differs from existing cross-cultural or Islamic bioethics models
  - Whether similar integrative models exist in other jurisdictions (e.g., Malaysia, UAE, or broader Muslim-majority contexts)
- The claim of originality (Saudi-adapted operational toolkit) is valid, but it should be **more explicitly benchmarked against prior studies**.

This would strengthen the manuscript’s contribution to **global comparative ethics discourse**, not only the Saudi context.

 5. Operational Framework: From Normative to Applied

 The proposed “Code of Conduct” and decision rules are among the strongest parts of the manuscript. However, their **practical applicability could be further enhanced**:

- The framework would benefit from:
  - One or two **worked examples or hypothetical scenarios** demonstrating how the decision rules operate in practice
  - Clarification on **implementation feasibility** in real institutional settings (e.g., resource constraints, training gaps)
- At present, the model is conceptually robust but remains somewhat **ideal-typical rather than practice-tested**.

6. Treatment of Digital Ethics

 The discussion on tele-mental health and digital boundaries is timely and relevant. However:

- It could be strengthened by:
  - Engaging more deeply with **recent empirical literature on digital therapeutic relationships**
  - Addressing **cross-border data governance issues**, especially given the global nature of telepsychology
- The manuscript correctly frames digital contact as a “boundary accelerant” , but the analysis could move further toward **regulatory foresight and future risks**.

7. Redundancy and Conceptual Repetition

 Several core concepts (e.g., fiduciary asymmetry, slippery slope, boundary crossings vs. violations) are repeated across sections. While important, this repetition:

- Reduces analytical density
- Creates an impression of conceptual inflation rather than progression

Streamlining these discussions would allow more space for **deeper critical engagement**.

 8. Normative Positioning and Tone

 The manuscript occasionally adopts a **prescriptive tone** (e.g., “must,” “should”) without always distinguishing between:

- Descriptive analysis (what is)
- Normative argument (what ought to be)

A clearer separation between these layers would improve academic rigor and avoid the impression of **policy advocacy without sufficient argumentative grounding**.

Is the work clearly and accurately presented and does it cite the current literature?

Yes

If applicable, is the statistical analysis and its interpretation appropriate?

Not applicable

Are all the source data underlying the results available to ensure full reproducibility?

Yes

Is the study design appropriate and is the work technically sound?

Yes

Are the conclusions drawn adequately supported by the results?

Yes

Are sufficient details of methods and analysis provided to allow replication by others?

Yes

Reviewer Expertise:

islamic law; family law; cultural studies

I confirm that I have read this submission and believe that I have an appropriate level of expertise to confirm that it is of an acceptable scientific standard, however I have significant reservations, as outlined above.

# Response

**Published:** 2026-05-18

**Authors:** Hajed A. Alotaibi

**Affiliations:** Majmaah University, Saudi Arabia

Thank you for your valuable comments, informative guidance. Here are our replies to the reviewer 2 comments: Strengthen the analytical contribution (tensions, prioritization, conflict resolution)

 Response: Thank you for this valuable suggestion. We have made the analytical contribution explicit by adding a dedicated conflict-resolution hierarchy and tie-break tests. Specifically, we added a subsection “Conflict resolution and prioritization rules” in Section 2.5 and a bridging sentence in Section 6 to signal that each decision rule applies this hierarchy. Manuscript insertions: • Section 2.5 (after the three constraints) — new subsection: Conflict resolution and prioritization rules. When Sharia-based norms, Saudi regulations, and global clinical ethics diverge, we adjudicate as follows: (1) Binding Saudi law and duly issued implementing regulations define minimum legal compliance; (2) Sharia maxims (harm prevention, bounded necessity, proportionality, dignity, and amānah) constrain ethically acceptable options within the legal space; (3) Clinical evidence and professional standards operationalize the least-restrictive, risk-minimizing course that preserves therapeutic integrity. Tie-break tests: demonstrable harm-reduction prevails over speculative benefits; necessity is bounded to the minimum effective action; and any derogation from default boundaries requires documentation and oversight. • Section 6 (end of opening paragraph) — add: For each decision rule, we apply the hierarchy (law → Sharia constraints → clinical proportionality) to avoid premature harmonization. Methodological rigor and justification (operationalizing integration; source selection)

 Response: We agree and have clarified both source selection/weighting and how conflicts are adjudicated. We added “Source selection and weighting” to Section 2.1 and an “Adjudication workflow” to Section 2.5. Manuscript insertions: • Section 2.1 (just before 2.2) — new paragraph: Source selection and weighting. We prioritized: (a) binding Saudi legal texts and implementing regulations; (b) national professional codes (SCFHS), MOH standards, and SDAIA/PDPL materials; (c) high-impact peer-reviewed clinical ethics literature and telepsychology guidance (JTF); and (d) Islamic bioethics/usul references directly addressing harm, amānah, maqāṣid, and sadd al-dharā’iʿ. Inclusion criteria: public accessibility, direct boundary relevance, and normative authority; exclusion: opinion pieces lacking relevance or provenance. Weighting favored recency for digital ethics, legal force for governance texts, and methodological standing for clinical sources. • Section 2.5 (append at end) — new lines: Adjudication workflow. On conflict: confirm legal minima; screen options via Sharia maxims (necessity measured by extent; no harm/reciprocating harm; dignity); then apply clinical proportionality (least-restrictive, auditable, role-limited). We document the path to ensure reproducibility. Depth of Sharia engagement (usul pathways; madhhab stance) Response: We expanded our usul grounding and clarified our jurisprudential stance. We added a “Methodological note on usul and madhhab stance” to Section 2.3 and linked specific qawāʿid in Sections 4.2 and 4.3. Manuscript insertions: • Section 2.3 (near the beginning) — new paragraph: Methodological note on usul and madhhab stance. We adopt a general Sunni usul approach, relying on widely accepted qawāʿid fiqhiyyah (e.g., lā ḍarar wa lā ḍirār; al-ḍarūrāt tubīḥ al-maḥẓūrāt with al-ḍarūrah tuqaddar bi-qadrihā; sadd al-dharā’iʿ) and the maqāṣid hierarchy (ḥifẓ al-nafs, al-ʿaql, al-ʿirḍ). Where juristic disagreement exists, we select positions compatible with the Saudi legal order that maximize harm-prevention and dignity while bounding necessity. This remains normative integration for policy design, not fatwa issuance. • Section 4.2 (add final sentence): This application relies on al-ḍarūrah tuqaddar bi-qadrihā to prevent over-expansion of exceptions. • Section 4.3 (add final sentence): The consent-integrity duty reflects amānah and sadd al-dharā’iʿ by blocking pathways that predictably induce dependency or coercion. Contextualization within existing literature (comparative positioning)

 Response: We added a comparative positioning paragraph at the end of the Introduction and a distinctiveness sentence in the Conclusion, benchmarking our Saudi-operational framework against cross-cultural Islamic bioethics. Manuscript insertions: • Introduction (final paragraph) — new text: Position within comparative ethics. Prior Islamic bioethics toolkits emphasize high-level principles; few translate them into a Saudi-operational boundary code aligned simultaneously with SCFHS, MOH standards, SDAIA/PDPL, Anti-Harassment Law, and telepsychology guidance. Jurisdictions such as Malaysia and the UAE offer pertinent precedents, yet lack a Saudi-calibrated operationalization that couples role-based access controls, digital response-window policies, and audit-ready templates. Our framework closes this gap by turning convergent principles into reproducible governance procedures. • Section 9 (final paragraph, add sentence at end): Distinct from cross-cultural models, this framework is jurisdiction-calibrated for Saudi governance (SCFHS, MOH, SDAIA) and embeds digital-era safeguards (authorized channels, logging, PDPL-consistent minimization) directly into boundary ethics. Operational framework: worked examples and feasibility

 Response: We have added one page of worked examples and a feasibility note on staged implementation. Manuscript insertions: • New Section 6.6 — insert after 6.5: 6.6 Worked examples. Example A (after-hours WhatsApp message from a distressed client): Apply the digital-policy rule—acknowledge only via authorized channel; redirect to the crisis pathway; document the contact; review in-session; reiterate response-window policy to reduce dependency (law → PDPL; Sharia → sadd al-dharā’iʿ; clinical → least-restrictive). Example B (dual role in a tight-knit community): If unavoidable, enact role-limits consent, an information firewall, early supervision, and referral at the earliest feasible juncture; document safeguards. This sequence upholds legal compliance, amānah, and proportionality while preserving continuity of care. • Section 8 (insert as first paragraph): Feasibility note. The framework supports incremental deployment: (1) adopt the short documentation templates; (2) designate a safeguarding/ethics lead; (3) publish authorized digital channels and response-window policy; (4) run brief, scenario-based refreshers. These steps are low-cost but yield immediate gains in auditability. Digital ethics depth (recent literature; cross-border governance)

 Response: We expanded digital sections to address cross-border processing and vendor diligence under PDPL. Manuscript insertions: • Section 5.4 (append at end): Cross-border risk. Tele-tools may route data through non-local clouds. PDPL and sectoral guidance require controller diligence on data localization or lawful transfer mechanisms and third-country adequacy. Boundary policies should incorporate vendor assessments and a register of cross-border transfers. • Section 6.5 “Effective policy factors” (add new item 6): Cross-border data controls: disclose if platforms involve extra-territorial processing; ensure a lawful transfer basis and record it; prefer Saudi-hosted or demonstrably PDPL-compliant services for therapy content. Redundancy and conceptual repetition

 Response: We streamlined Sections 3.1–3.4 to single concise definition paragraphs and replaced later repetitions with cross-references (“as defined in Section 3”), freeing space for deeper analysis. Manuscript changes: Condensed 3.1–3.4; inserted cross-references in later sections in place of restatements. Normative positioning and tone

 Response: We now explicitly distinguish descriptive (what is) from normative (what ought to be) in Section 2.1, and we limited prescriptive wording primarily to the Code of Conduct and decision rules, using “we recommend/this framework requires within policy” elsewhere. Manuscript insertions: • Section 2.1 (final sentence): We distinguish descriptive analysis of current Saudi law/policy from normative recommendations; prescriptive language is confined to the proposed Code and decision rules.
